# Epidemic Influenza

**Published:** 1891-07

**Authors:** R. A. Chudleigh


### EPIDEMIC INFLUENZA.

For about thirty years I have taken especial interest in all the influenzas, whether of Pitcairn or of the Chatham Islands, or of St. Kilda, or the great wave of epidemic which was sweeping round the world about this time last year. My own conviction is that there is nothing which one can take either as a prophylactic or a cure which exerts any material influence on the disease. Or, if one must take something, let it be great care ; care, I mean, to avoid chills, ulcerations, over-heat, and everything calculated to produce inflammation or a feverish condition, either localized as in a sore throat, or general as in a universal chill. For I have a steadily growing belief that influenza is a microbic disorder, whose microbes do not readily take hold on the human system at its normal temperature, but if there be even a slight degree of fever, either local or general, the germs find a readier entrance, and this idea is strengthened by the fact that the animals which are most susceptible of it, and which feel its approach much earlier than men do, are all animals whose blood-heat is normally higher than that of man. There is a theory now afloat that influenza is due to excess of ozone in the air. It is true that a person who sits in the vicinity of an electric machine which is generating ozone gets something very similar to influenza. But if the theory be sound, the best way to avoid influenza would be to live near a drain ; for the good of ozone, and the only good from the sanitary standpoint, is that it neutralizes putrescible matter. So, if "Fenman" lives in the fens, there should be no such visitation as an influenza epidemic—ozone and the fen exhalations should destroy each other.—R. A. CHUDLEIGH, *in Farm and Home, Eng.*

